# Ncf1 (p47phox) Is Essential for Direct Regulatory T Cell Mediated Suppression of CD4+ Effector T Cells

**DOI:** 10.1371/journal.pone.0016013

**Published:** 2011-01-06

**Authors:** Olga Efimova, Philippe Szankasi, Todd W. Kelley

**Affiliations:** 1 Department of Pathology, University of Utah, Salt Lake City, Utah, United States of America; 2 ARUP Laboratories, Salt Lake City, Utah, United States of America; New York University, United States of America

## Abstract

**Background:**

Multiple mechanisms have been advanced to account for CD4+FOXP3+ regulatory T cell (Treg)-mediated suppression of CD4+ effector T cells (Teffs) but none appear to completely explain suppression. Previous data indicates that Tregs may affect the microenvironment redox state. Given the inherent redox sensitivity of T cells, we tested the hypothesis that oxidants may mediate the direct suppression of Teffs by Tregs.

**Methodology/Principal Findings:**

Tregs and Teffs were isolated from the spleens of wild type (WT) C57BL/6 mice or Ncf1(p47phox)-deficient C57BL/6 mice which lack NADPH oxidase function. Teffs were labeled with CFSE and co-cultured with unlabeled Tregs at varying Treg:Teff ratios in the presence of anti-CD3/CD28 coated beads for 3 days in suppression assays. Treg-mediated suppression was quantified by flow cytometric analysis of CFSE dilution in Teffs. The presence of the antioxidants n-acetylcysteine (NAC) or 2-mercaptoethanol or inhibitors of NADPH oxidase (diphenyleneiodonium and VAS-2870) resulted in reduced WT Treg-mediated suppression. The observed suppression was in part dependent upon TGFβ as it was partially blocked with neutralizing antibodies. The suppression of Teff proliferation induced by exogenous TGFβ treatment could be overcome with NAC. Ncf1-deficient Teff were slightly but significantly less sensitive than WT Teff to suppression by exogenous TGFβ. Ncf1-deficient Tregs suppressed Ncf1-deficient Teff very poorly compared to wild type controls. There was partial but incomplete reconstitution of suppression in assays with WT Tregs and Ncf1-deficient Teff.

**Conclusions/Significance:**

We present evidence that NADPH oxidase derived ROS plays a role in the direct Treg mediated suppression of CD4+ effector T cells in a process that is blocked by thiol-containing antioxidants, NADPH oxidase inhibitors or a lack of *Ncf1* expression in Tregs and Teffs. Oxidants may represent a potential new target for therapeutic modulation of Treg function.

## Introduction

Numerous factors have been reported to account for CD4+FOXP3+ regulatory T cell (Treg)-mediated immune suppression including those that are cytotoxic such as perforin and granzyme B [1] and those that suppress proliferation and cytokine secretion in target cells. The latter category includes factors such as IL-10, TGFβ and CTLA-4 and mechanisms such as suppression of IL-2 production by target cells or Il-2 sequestration by Tregs [2]. Some of these mechanisms are active in *in vitro* suppression assays, where Tregs and CD4+ target cells are co-cultured in the presence of stimuli, whereas others have been identified primarily in *in vivo* models. The ultimate contribution of any particular mode of suppression *in vivo* is unclear but is the subject of widespread inquiry. Overall, the existing data is somewhat contradictory but indicates that there are multiple and likely-context dependent pathways whereby Tregs suppress the activation of CD4+FOXP3- T effector cells (Teffs). The existing literature indicates that this occurs by both direct mechanisms, which can be measured in *in vitro* co-culture assays, and by indirect mechanisms where Tregs modulate the activity of dendritic cells and macrophages that, in turn, affect suppression. The latter scenarios are likely best evaluated in *in vivo* models.

One of the major suppressive factors employed by Tregs is TGFβ. TGFβ is present primarily in a membrane bound form on Tregs [3], [4], [5]. Neutralizing antibodies to TGFβ block Treg-mediated suppression of Teffs *in vitro* [4], [5] and targeted deletion of the TGFβ receptor renders Teffs non responsive to Treg mediated suppression *in vivo* [6]. In murine models with T cell specific TGFβ deletion, there is severe autoimmune disease that results in death only 1–2 weeks after birth [7]. TGFβ appears particularly important for the suppression of autoimmune colitis [8]. The mechanism by which TGFβ suppresses T cell activation remains mostly unresolved but likely involves a variety of pathways [9]. TGFβ has numerous other functions as well. Together with IL-2, it is important for the peripheral differentiation of suppressive, induced Tregs that also express the Treg specific transcription factor FOXP3 [10]. In non-immune cell types including hepatocytes and myofibroblasts, TGFβ appears to activate pathways that, at least in part, act via upregulation of the prooxidant enzyme complex NADPH oxidase [11], [12], [13]. In accordance with this, various effects of TGFβ may be suppressed by treatment with antioxidants such as n-acetylcysteine (NAC) [14]. This is illustrated by the inhibition of TGFβ -mediated epithelial-mesenchymal transition of alveolar epithelial cells by NAC both *in vivo* and *in vitro* [15]. The possibility that NAC modulates TGFβ signaling in T cells has, to our knowledge, not been investigated.

Cysteine is important for T cell proliferation and activation and is a precursor to glutathione (GSH). Depletion of intracellular GSH with L-buthionine sulfoximine (BSO), an inhibitor of GSH synthesis, results in suppression of T cell proliferation [16]. T cells are particularly sensitive to cysteine depletion in the extracellular milieu as they are unable to take up cystine, the major extracellular form. However, dendritic cells secrete cysteine and in this way regulate the proliferative potential of local T cells by controlling cysteine availability [17]. Recent data suggests that Tregs are able to suppress the secretion of cysteine into the microenvironment by dendritic cells to indirectly suppress Teff proliferation via cysteine depletion [18]. Myeloid derived suppressor cells (MDSCs) also deprive T cells of cysteine and thus appear to act in a similar fashion [19]. Interestingly, T cells are also very sensitive to the levels of reduced cell surface thiols, or the cell surface redox state, and their proliferation and activation can be increased by increasing surface –SH groups with NAC or glutathione [20]. Cellular redox balance may be influenced by the activity of NADPH oxidase, a major intracellular source of oxidants and a complex that is present in T cells [21]. NADPH oxidase is comprised of multiple subunits, two of which, Ncf1 (p47^phox^) and NOX2 (gp91^phox^), have been implicated as necessary for sustained reactive oxygen species (ROS) production in T cells [21]. ROS can be detected upon T cell receptor (TCR) stimulation [22], [23] and functions to suppress signaling effectors, such as ERK [24]. Thus, in T cells ROS may, at least in certain circumstances, act as a negative regulator of activation. The possibility that Tregs directly induce oxidant stress in Teffs has not widely investigated as a mechanism of suppression.

In light of these previous findings, we evaluated the role of ROS in direct Treg-mediated suppression of Teffs using antioxidants, NADPH oxidase inhibitors and T cells from Ncf1-deficient (*Ncf1^−/−^*) mice that lack a functional NADPH oxidase complex due to a substitution mutation that results in aberrant *Ncf1* gene splicing. This mutation results in a lack of detectable Ncf1 protein by western blotting and a lack of NADPH oxidase function [25]. Our studies showed that Treg suppression of Teffs was partially dependent on TGFβ and could be reduced or blocked by NAC, 2-mercaptoethanol (2-ME) or inhibitors of NADPH oxidase (diphenyleneiodonium [26] and VAS-2870 [27]). Furthermore, experiments performed in Ncf1-deficient cells demonstrated an essential role for a functional NADPH oxidase complex in *both* Tregs and target Teffs for optimum suppression. These findings suggest that oxidants mediate an unexplored pathway of direct Treg-mediated suppression of Teffs. Furthermore, they suggest that targeted antioxidant therapy may be clinically helpful in modulating the suppressive activity of Tregs *in vivo* in circumstances, such as the tumor microenviroment, where blocking the function of Tregs may be beneficial.

## Materials and Methods

### Ethics Statement

All experimental procedures were approved by the University of Utah Institutional Animal Care and Use Committee (protocol #08-07012).

### Mice

The mutation that resulted in the Ncf1 (p47^phox^)-deficient mouse strain (B6(Cg)-*Ncf1^m1J/^*J) arose spontaneously in C57BL/6 mice with *Lepr* mutations and has been described [25]. The original *Lepr* mutations have been bred out by crossing onto wild type C57BL/6 mice. This strain and wild type C57BL/6 mice were both purchased from Jackson Laboratory (Bar Harbor, ME). Mice were maintained in a pathogen free environment in temperature-controlled conditions under a 12-hour light/dark cycle. Animals were used in experiments at between 6 and 14 weeks of age.

### Reagents and cell culture

RPMI 1640 medium (HyClone, Logan, UT) containing 10% fetal calf serum (HyClone) was used for cell culture. Cultures were maintained in a 37°C incubator containing 5% CO_2_. Beads coated with anti-mouse CD3/CD28 were from Invitrogen (Carlsbad, CA). Sterile, preservative-free n-acetylcysteine solution (20%) was from American Regent (Shirley, NY). 2-mercaptoethanol and dimethyl sulfoxide (DMSO) were from Sigma-Aldrich (St. Louis, MO). Where indicated, recombinant human TGFβ1 (R&D Systems, Minneapolis, MN) or anti-mouse TGFβ1 blocking antibodies (R&D Systems) were added to T cell cultures. L-buthionine sulfoximine was from Sigma, the flavoprotein inhibitor diphenyleneiodonium chloride and the NADPH oxidase inhibitor VAS-2870 [27] were both from Enzo Life Sciences (Plymouth Meeting, PA).

### Flow cytometry

Flow cytometric immunophenotyping was performed on a 4-color Accuri C6 flow cytometer (Accuri Inc, Ann Arbor, MI). Data was analyzed using FCS Express software (De Novo Software, Los Angeles, CA). Anti-mouse CD25-PE was from Miltenyi Biotec (Auburn, CA), anti-mouse CD4-FITC (clone RM4-5) and anti-mouse FOXP3-APC (clone FJK-16s) were both from eBioscience (San Diego, CA). Permeabilization was performed using a FOXP3 fixation/permeabilization kit, also from eBioscience. Flow cytometric sorting was performed on a FACSAria II sorter (BD Biosciences, San Jose, CA).

### T cell isolation

Spleens were removed from sacrificed mice and macerated then strained with sterile cell strainers (BD Biosciences, Bedford, MA) to create a single cell suspension. Red blood cells were subsequently lysed with RBC lysis buffer (eBioscience). Samples were washed and CD4+CD25+FOXP3+ Tregs were isolated based on their surface expression of CD25 using a magnetic bead based isolation kit (Miltenyi Biotec, Inc.) per the manufacturer's instructions. The CD4+CD25- fraction was used as Teff cells.

### *In vitro* suppression assays

**Figure 1:**
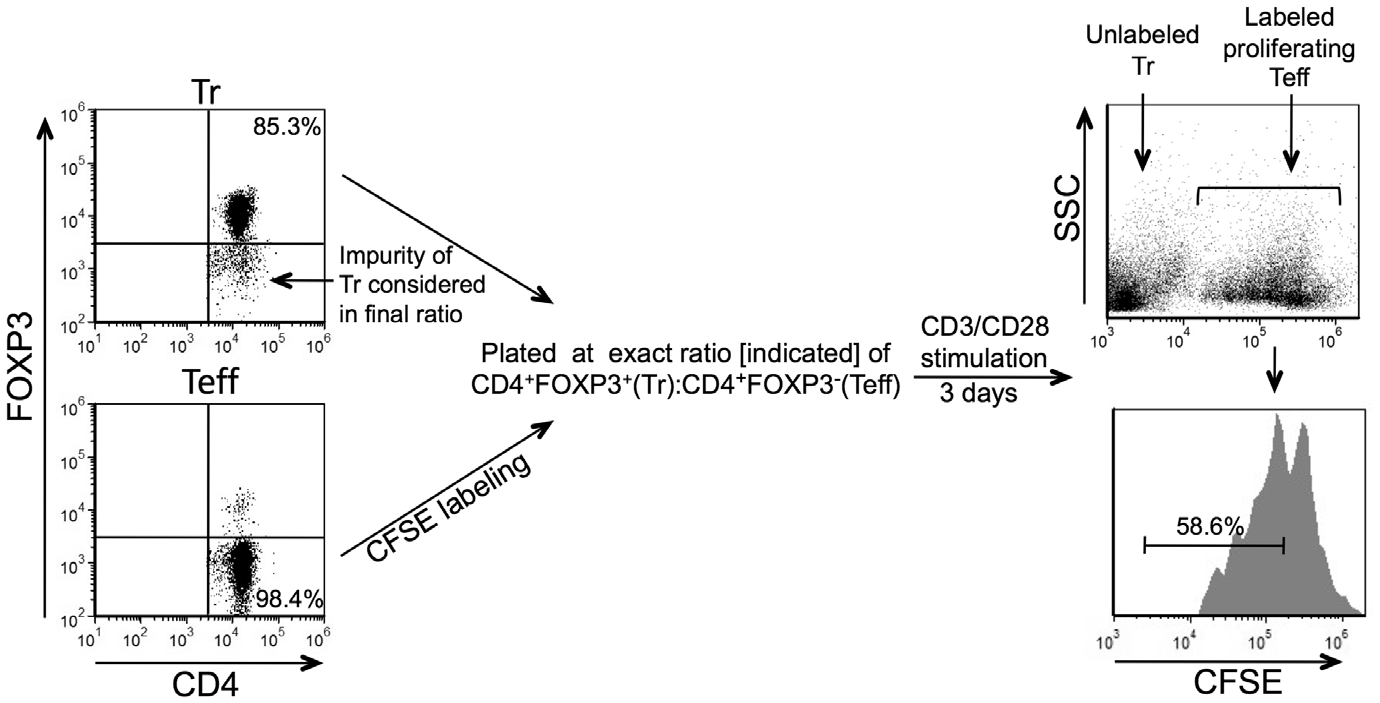
Outline of strategy for performing *in vitro* suppression assays to measure Treg suppression of Teff. Treg and Teff subsets were isolated from mouse spleens and evaluated by flow cytometry prior to co-plating. The exact number of CD4+FOXP3- cells co-purifying with Tregs (usually 10–15%; see arrow) was taken into consideration for plating so that the final CD4+FOXP3+ Treg:CD4+FOXP3- Teff ratio was exactly 1∶1 (or 2∶1 or 1∶2 as indicated). The low and consistent number of contaminating CD4- cells in the isolates was not considered in the ratio and are not shown in the dot plots. Prior to co-plating, the Teff subset was labeled with CFSE. The Treg:Teff mixtures were then added together to round bottom 96 well plates and stimulated with anti-CD3/CD28 coated beads at a bead:cell ratio of 1∶1, in triplicate, for three days. Percent suppression was calculated by comparing the proliferation of Teffs co-plated with Tregs to Teffs plated in the same conditions without Tregs.

After isolation as above, a portion of the Treg and Teff samples was subjected to flow cytometric evaluation for expression of CD4, CD25 and FOXP3 prior to mixing for suppression assays. Because a small percentage of CD4+CD25+ T cells are not Tregs and do not express FOXP3, the isolated Treg samples were never completely pure, based on FOXP3 expression, and ranged from approximately 80%–85% FOXP3 positive. To ensure optimal reproducibility of the suppression assays, the degree of purity of the Treg samples was taken into account when the cells were plated so that the final ratio of CD4+FOXP3+ Tregs to CD4+FOXP3- Teff was as close as possible to 1∶1, 1∶2 or 2∶1 as indicated in the figures. The consistent and low percentage of CD4- cells that co-purified with the CD4+ cells was not considered in the final ratio. Prior to co-plating Tregs and Teffs, the CD4+FOXP3- Teff cells were labeled with carboxyfluorescein diacetate, succinimidyl ester (CFSE; Invitrogen) by incubating in 1 µM CFSE in phosphate buffered saline at 37°C for 3.5 mins. CFSE stock solutions were diluted in DMSO prior to use. Samples were then placed in RPMI + 10% FCS and incubated on ice for 10 minutes then washed 2 times. Treg:Teff mixtures or control samples consisting of Teffs alone were plated in RPMI + 10% FCS in 96 well round bottom plates (Corning Life Sciences, Lowell, MA) in triplicate. All wells contained an equal final number of total CD4+ T cells. Anti mouse-CD3/CD28 coated beads (Invitrogen) were added to a final ratio of 1 bead per cell. Samples were then incubated in a 37°C incubator for 3 days. The proliferation of CFSE labeled Teffs was subsequently evaluated by a flow cytometric quantification of CFSE dilutional staining as described [28]. Percent suppression was calculated using proliferation measurements and the following formula: suppression (%)  =  (1 - Teffs in Treg:Teff/Teffs alone) ×100. A general outline of the suppression assay protocol is shown in **Figure 1**.

### Quantitative RT-PCR analysis of *Ncf1, Ncf2 and gp91^phox^* expression

C57BL/6 mouse CD4+CD25- T cells were purified by flow cytometric sorting (>98% pure) then stimulated with CD3/CD28 beads +/− TGFβ (10 ng/mL) for one or two days as indicated. Total RNA was isolated from samples using the RNeasy mini kit (Qiagen Inc, Germantown, MD) per the manufacturer's instructions. The levels of mRNA for *Ncf1*, *Ncf2*, and *gp91^phox^* were determined by quantitative RT-PCR. Briefly, 0.4–0.8 µg total RNA was reverse transcribed using random hexamer primers and the Superscript III First Strand cDNA system for RT-PCR (Invitrogen) and the resulting cDNA was brought to 40 µL with water. RT-PCR was carried out on a LightCycler instrument (Roche Diagnostics, Indianapolis, IN) using the LightCycler FastStart DNA Master SYBR Green kit (Roche Diagnostics). Duplicate 20 µL PCR reactions contained 2 µL cDNA, 1X master mix, 3 mM MgCl_2_ and 0.5 µM each primer for *Ncf1* and *Ncf2*; 3 mM MgCl_2_ and 0.25 µM each primer for *gp91^phox^*; and 4 mM MgCl_2_ and 0.5 µM each primer for the house keeping gene *Actb*. PCR primer sequences were as follows (all 5′-3′): Ncf1 forward – GAGCCGCTGAGAGTCGCCAG; Ncf1-reverse – CCACGTCTCCGGTTGCCACG; Ncf2-forward – CCTCAGTCGCAGCCCCAGGA; Ncf2-reverse – GCCAAGCCGCGTCTCCATGA; gp91^phox^-forward - CCGGGATTGGAGTCACGCCC; gp91^phox^-reverse – AGGCATGCGTGTCCCTGCAC; ActB-forward - AGCACAGCTTCTTTGCAGCTCCTTC; ActB-reverse - CTGGGCCTCGTCACCCACAT. The cycling conditions were 95° for 10 minutes followed by 50 cycles of 95° for 2 seconds, 64° (65° for *Ncf2*) for 2 seconds and 72° for 2 seconds and a single fluorescence acquisition. The purity of the PCR products was confirmed by melting analysis: samples were heated to 95° then immediately brought to 65° for 15 seconds followed by heating to 95° at 0.1° per second with continuous fluorescence acquisition. *Ncf1*, *Ncf2*, and *gp91^phox^* values were normalized to those of *Actb* and expressed relative to flow sorted but untreated cells at day 0 as described in ref [29]. Amplification efficiencies for each primer pair were greater than 95%.

### Measurement of intracellular ROS

Intracellular ROS was measured by flow cytometry with 5-(and-6)-chloromethyl-2′,7′-dichlorodihydrofluorescein diacetate, acetyl ester (DCFDA; Invitrogen). Briefly, samples were stimulated in the indicated conditions for 24 hours then washed without removing the CD3/CD28 beads and resuspended in PBS with 2 µM DCFDA. Each sample was then incubated with DCFDA for exactly 15 minutes at 37°C. DCFDA fluorescence was then immediately assessed by flow cytometry.

### Statistics

Pairwise statistic analysis using the Student's t-test was performed using GraphPad Prism version 5.0 (GraphPad Software, San Diego, CA).

## Results

### Thiol-bearing antioxidants and NADPH oxidase inhibitors block or reduce direct Treg mediated suppression of CD4+ effector T cells

**Figure 2:**
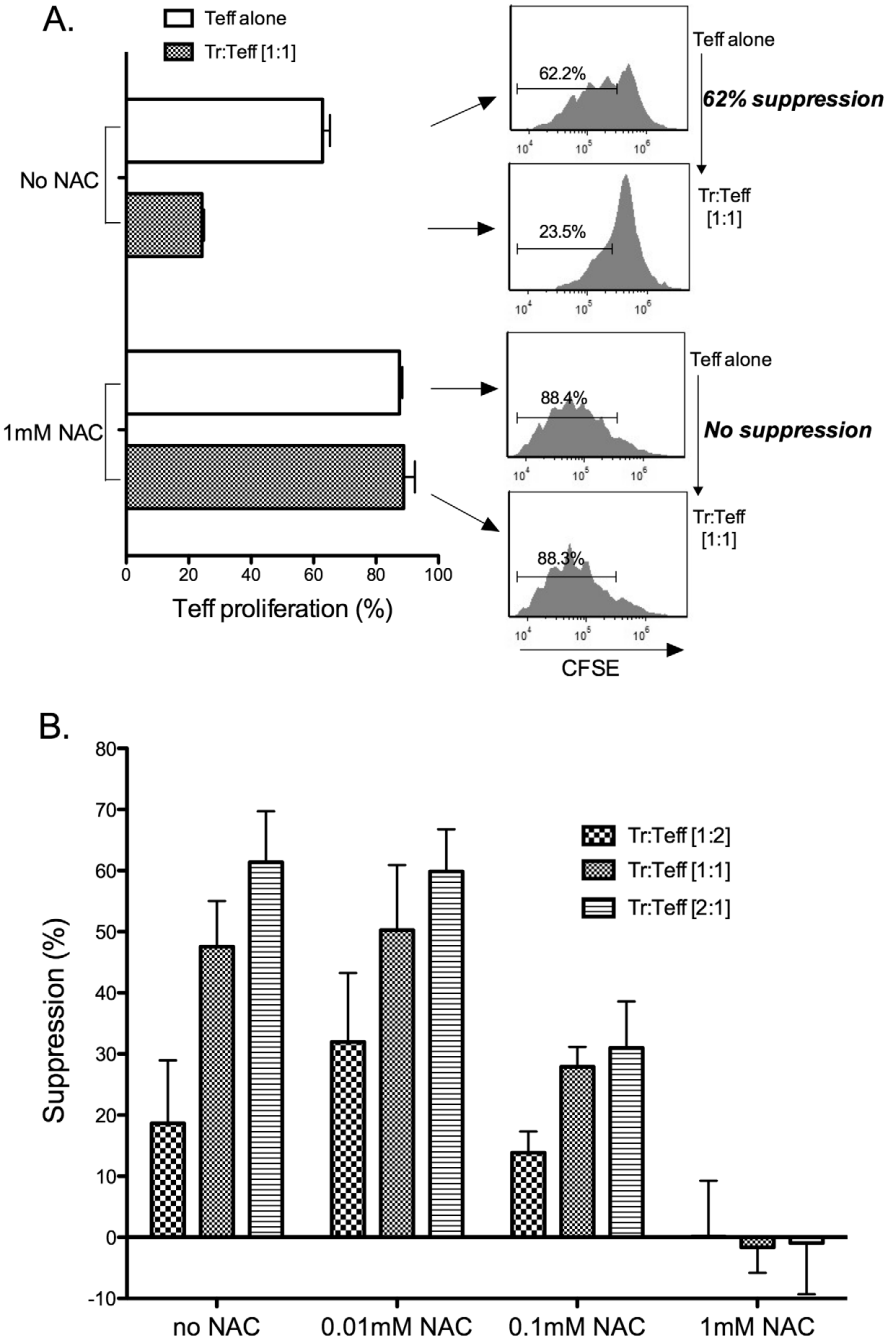
The antioxidant n-acetylcysteine (NAC) blocks direct Treg mediated suppression of Teffs in a dose dependent fashion. Panel (A) shows the proliferation of CD3/CD28 stimulated Teffs (CFSE labeled) plated alone (white bars) or Teff plated at a 1∶1 ratio with Tregs (patterned bars) for 3 days, with and without 1 mM NAC. Data showing flow cytometric evaluation of CFSE staining with the calculated suppression (%) is shown at right. This data is representative of that seen in more than 3 experiments. Panel (B) shows Treg-mediated suppression of Teff under conditions of CD3/CD28 stimulation for three days in increasing concentrations of NAC (0.01 mM, 0.1 mM and 1 mM) with increasing Treg (labeled Tr in figure) to Teff ratios of 1∶2, 1∶1 and 2∶1. The data represents a compilation of data from three identical experiments each performed in triplicate and each yielding similar findings.

In order to assess the potential contribution of ROS to Treg suppression of Teffs we performed conventional Treg suppression assays, using wild type cells and a 1∶1 ratio of Tregs to Teffs, in the presence of the antioxidant NAC to block ROS. Surprisingly, we found that it completely prevented Treg-mediated suppression (**Figure 2A**). Identical control assays performed in the absence of NAC confirmed that the Tregs were suppressive, typically generating approximately 60% suppression of Teff proliferation when compared to Teffs cultured alone.

We next performed dose response experiments using varying ratios of Tregs to Teffs (1∶2, 1∶1 and 2∶1) and increasing doses of NAC (0, 0.01 mM, 0.1 mM and 1 mM) in suppression assays (**Figure 2B**). In the absence of NAC, suppression of Teffs increased with increasing Treg:Teff ratios. The lowest concentration of NAC (0.01 mM) had no effect on suppression at any ratio. At a concentration of 0.1 mM, NAC treatment resulted in significantly reduced suppression at 1∶1 (p = 0.01) and 2∶1 (p = 0.01) ratios of Tregs:Teff. The highest NAC concentration (1 mM) resulted in essentially absent suppression (p = 0.08 for 1∶2 ratio; p = 0.002 at 1∶1 ratio and p<0.001 at 2∶1 ratio). These studies indicate that a NAC concentration ≥0.1 mM in the extracellular milieu is sufficient to inhibit or block suppression.

**Figure 3:**
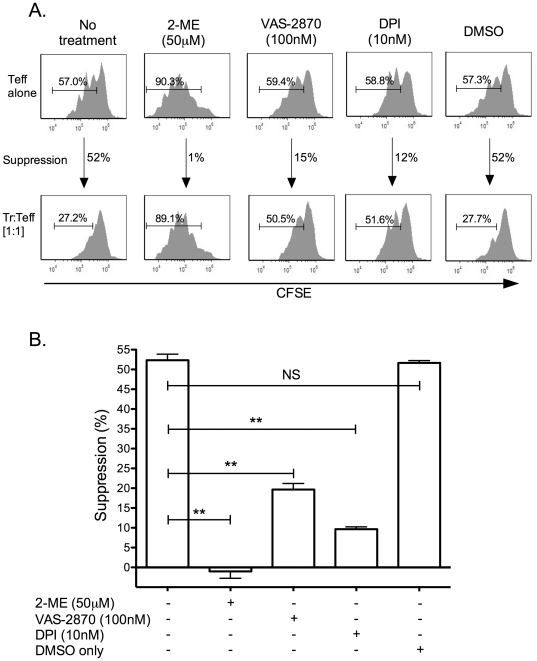
2-mercaptoethanol and inhibitors of NADPH oxidase (VAS-2870 and DPI) block or reduce direct Treg-mediated suppression of Teffs. Suppression assays were performed as outlined in the presence of 2-mercaptoethanol (2-ME) or the inhibitors of NADPH oxidase, VAS-2870 or diphenyleneiodonium (DPI) at the indicated concentrations, or DMSO alone as a solvent only control, or without any added agents as a positive suppression control. Panel (A) shows representative CFSE data from a single experimental trial. Panel (B) shows a compilation of all data from three separate experiments, each performed in triplicate, and each generating similar results (NS – not significant, ** p≤0.01).

To assess the specificity of the findings, we performed suppression assays in the presence of another thiol-bearing antioxidant compound, 2-ME (**Figure 3A/B**) and found that 2-ME augmented proliferation and completely blocked suppression. In order to assess whether or not the observed effects of antioxidants could be duplicated by inhibiting the production of intracellular oxidants by NADPH oxidase, we performed suppression assays in the presence of NADPH oxidase inhibitors VAS-2870 and diphenyleneiodonium and found that at very low concentrations (100 nM and 10 nM, respectively) there was a significant decrease in suppression (**Figure 3A/B**). However, in contrast to NAC and 2-ME, neither of the two inhibitors had any effect on augmenting the proliferation of Teffs alone (Figure 3A).

### Suppression by Tregs is partially dependent on TGFβ and n-acetylcysteine can overcome the suppressive effect of exogenous TGFβ1 on proliferation of isolated CD4+ effector T cells

**Figure 4:**
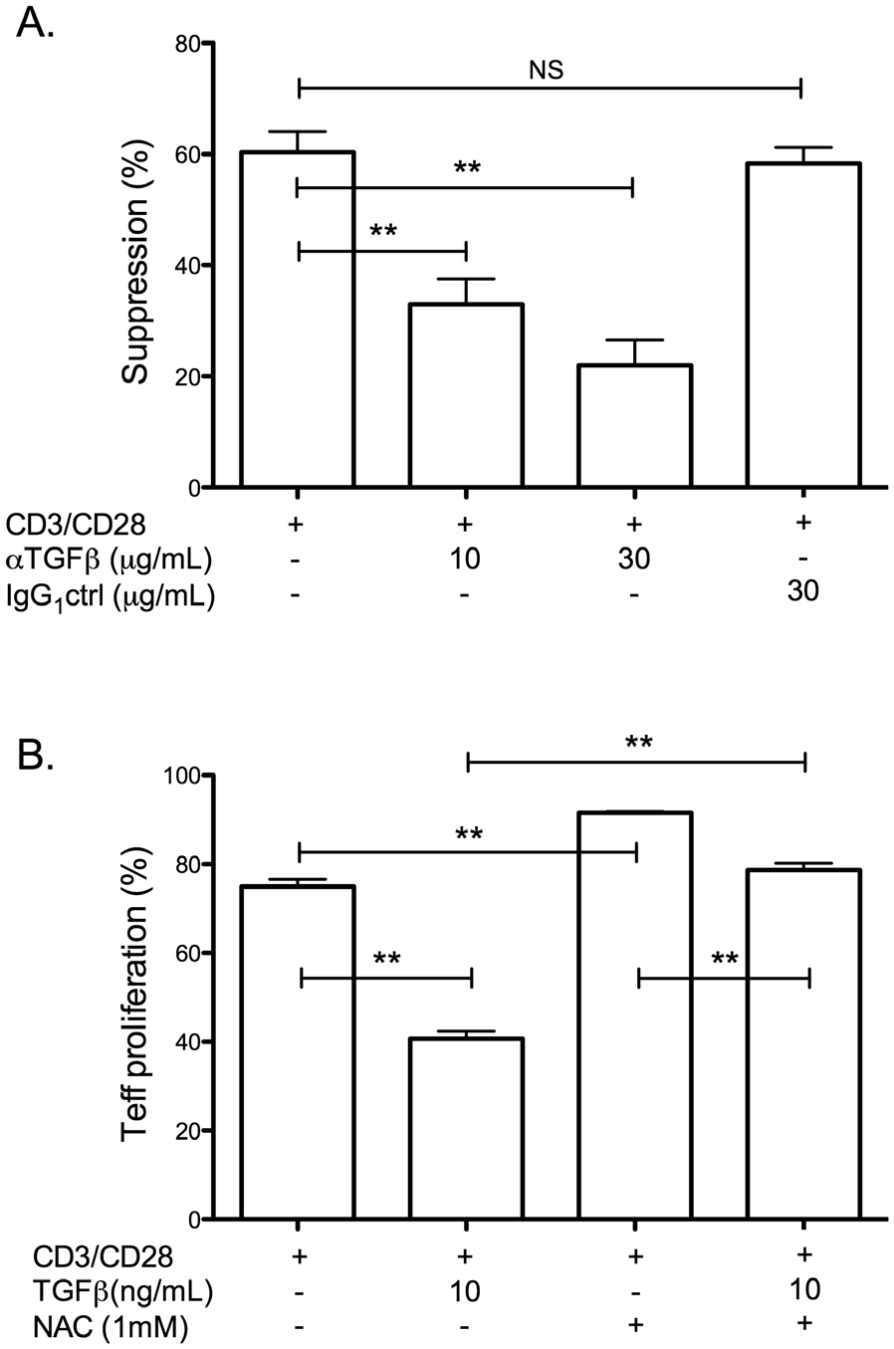
Suppression by Tregs is partially dependent on TGFβand the suppressive effects of exogenous TGFβ on Teff proliferation can be overcome with NAC. (A) Suppression assays were performed as outlined with a 1∶1 ratio of Tregs:Teff with and without TGFβ neutralizing antibodies at the indicated concentration, or nonspecific isotype control antibodies (IgG_1_), and anti-CD3/CD28 beads for 3 days. Similar results were obtained in at least three identical experiments, each performed in triplicate. The data shown represents a compilation of all of these experiments. (B) Purified, CFSE-labeled Teffs, without Tregs, were stimulated in the indicated conditions for 4 days with and without the addition of TGFβ (10 ng/mL) and NAC (1 mM). Proliferation was measured by flow cytometric evaluation of CFSE staining. The data shown is representative of three identical experiments (NS – not significant, ** p≤0.01).

In order to investigate the mechanism by which NAC overcomes the suppressive function of Tregs, it was necessary to identify the suppressive mediator or factor in the assay. We suspected, based on previous findings, that the effect could be at least be partly attributable to TGFβ. Therefore, we performed suppression assays in the presence of a TGFβ neutralizing antibody, or isotype control (**Figure 4A**). As before, suppression assays were performed at a 1∶1 ratio of Tregs: Teffs. Our observations confirmed the findings of others [4] and showed that neutralization of TGFβ partially but incompletely blocked suppression. This effect increased with increasing concentrations of neutralizing antibody but was completely absent with a nonspecific isotype control antibody, thus confirming the specificity. We next sought to determine whether or not the presence of NAC was sufficient to overcome the suppressive effects of exogenous TGFβ on the proliferation of CD3/CD28 stimulated Teff, in the absence of Tregs. We found that 10 ng/mL TGFβ resulted in a significant decrease in the proliferation of Teff but proliferation was restored by the addition of 1 mM NAC (**Figure 4B**).

### TGFβ augments intracellular ROS production in CD3/CD28 stimulated wild type but not Ncf1-deficient Teff

Because we had identified TGFβ as an important factor for the Treg-mediated suppression observed in our *in vitro* assays, and because suppression by Tregs and the suppressive effects of exogenously added TGFβ were both reversible by the antioxidant NAC, we were interested in determining intracellular ROS levels in TGFβ treated Teffs. To perform these experiments we utilized wild type (WT) Teffs and Teffs isolated from Ncf1-deficient mice that are unable to produce ROS via NADPH oxidase. The *Ncf1* (*p47^phox^*) gene encodes a component of the NADPH oxidase complex that is required for sustained ROS production in T cells in response to TCR triggering [21]. Ncf1-deficient mice have a syndrome similar to a human disorder arising from *Ncf1* mutations (chronic granulomatous disease; CGD) that can be attributed primarily to a lack of effective antimicrobial killing due to absence of NADPH oxidase function in phagocytes [30]. However, they also suffer from T cell mediated autoimmune arthritis [31] and therefore also mimic some of the associated autoimmune manifestations of human CGD.

**Figure 5:**
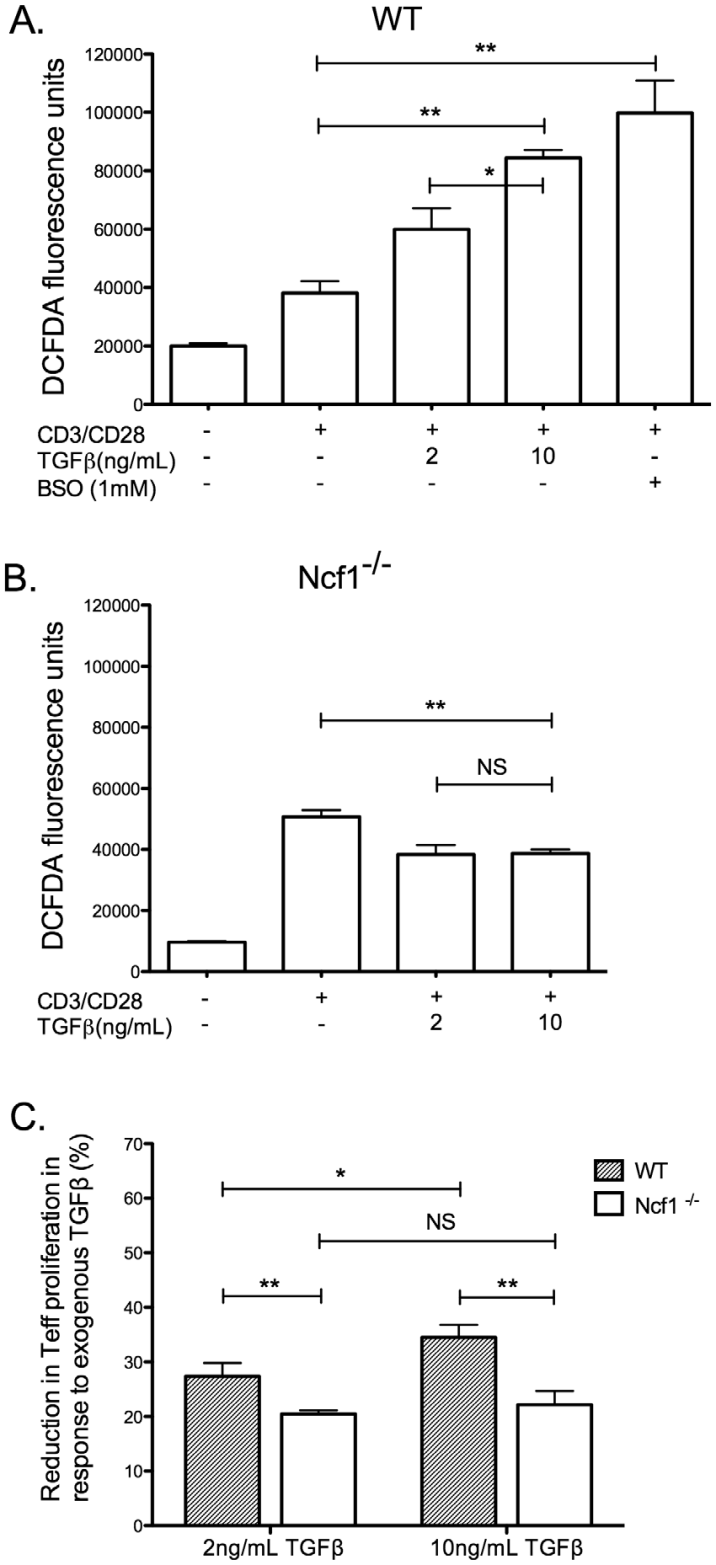
Treatment of Teffs with TGFβ results in a dose dependent elevation of intracellular oxidants. (A) Isolated, unlabeled Teffs, without Tregs, were cultured in the indicated conditions with exogenous TGFβ (2 and 10 ng/mL) or with the gamma-glutamylcysteine synthetase inhibitor buthionine sulfoximine (BSO; 1 mM) to deplete intracellular glutathione, and anti-CD3/CD28 beads for 24 hours. Subsequently, samples were labeled with the fluorescent, oxidant sensitive cell permeable indicator dye 5-(and-6)-chloromethyl-2′,7′-dichlorodihydrofluorescein diacetate, acetyl ester (DCFDA), each for exactly 15 minutes then immediately subjected to flow cytometric evaluation of DCFDA fluorescence. The data shown represents mean fluorescence intensity of DCFDA and is representative of three identical experiments, each performed in triplicate and each yielding similar results. (B) A similar experiment was performed using Teffs isolated from Ncf1-deficient mice. (C) CFSE labeled WT or Ncf1-deficient Teffs were stimulated as indicated for four days with anti-CD3/CD28 beads with and without exogenous TGFβ (2 and 10 ng/mL) then evaluated for proliferation by flow cytometric quantification of CFSE staining. The data shown represents the % reduction in proliferation of TGFβ treated Teffs vs untreated Teff samples and is representative of three identical experiments (NS – not significant, * p<0.05, **p≤0.01).

We evaluated intracellular ROS in samples subjected to CD3/CD28 stimulation in the presence of increasing concentrations of TGFβ. We also included control cells treated with L-buthionine sulfoximine (added to experiments with WT Teff only), an irreversible inhibitor of gamma-glutamylcysteine synthetase that depletes intracellular glutathione by blocking its synthesis and thus neutralizes the endogenous cellular antioxidant capacity. We assessed intracellular ROS with the cell permeable indicator dye DCFDA, an agent that fluoresces when oxidized and can be measured by flow cytometry in its oxidized state. We found that exogenous TGFβ resulted in augmented intracellular ROS in WT (**Figure 5A**) but not Ncf1-deficient Teffs (**Figure 5B**) versus that seen with CD3/CD28 stimulation without TGFβ. However, both groups exhibited similar increases in ROS in response to CD3/CD28 triggering alone and this suggests that sources of ROS other than NADPH oxidase are available during prolonged TCR signaling.

We next evaluated the ability of exogenous TGFβ to suppress proliferation of WT and Ncf1-deficient Teffs and found that there was a significant dose dependent reduction in proliferation of WT Teffs in response to TGFβ (**Figure 5C**). Ncf1-deficient Teffs also demonstrated reduced proliferation in the presence of TGFβ but there was no dose dependence and the reduction in proliferation was significantly less than that observed in WT cells. Together, the data indicate that Ncf1 is required for the TGFβ mediated augmentation of ROS. Furthermore, a lack of Ncf1 results in blunted, though not absent, suppression of proliferation in response to TGFβ.

### CD3/CD28 stimulation of Teffs results in upregulation of Ncf1 mRNA expression but not Ncf2 or gp91^phox^

**Figure 6:**
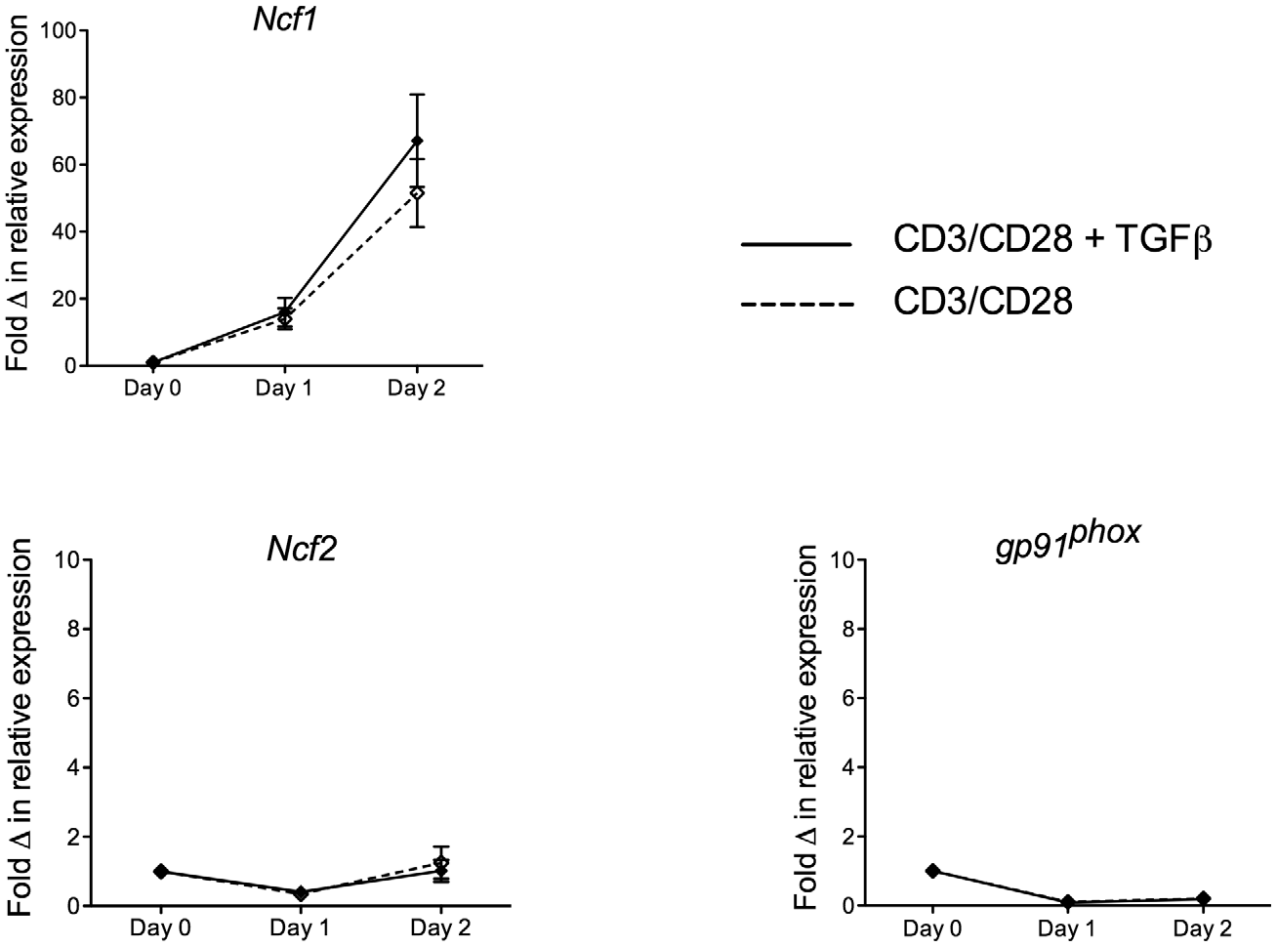
Expression of *Ncf1*, *Ncf2* and *gp91^phox^* in CD3/CD28 stimulated Teffs with and without TGFβ. Teffs were purified from WT C57BL/6 mouse spleen suspensions by flow cytometric sorting (>98% pure CD4+CD25- cells after sorting) then subjected to stimulation with CD3/CD28 beads with and without exogenous TGFβ (10 ng/mL) for 1 and 2 days. Total RNA was isolated from freshly sorted but unstimulated cells (Day 0) and from stimulated cells at day 1 and day 2. mRNA expression of three components of the NADPH oxidase complex, *Ncf1*, *Ncf2* and *gp91^phox^*, was measured by RT-PCR and normalized to expression of the housekeeping gene, β-actin. The data is graphed as fold-change in expression relative to Day 0 (arbitrarily defined as a relative expression of 1). The figure represents a compilation of data from three separate experiments.

Given the fact that we had noted a significant augmentation of intracellular ROS in the presence of TGFβ in WT Teff, we wondered if this effect was due to increased expression of components of the NADPH oxidase complex, *Ncf1*, *Ncf2* or *gp91^phox^*. To evaluate this possibility, Teffs were isolated by flow cytometric sorting of CD4+CD25- splenic T cells and stimulated with CD3/CD28 beads with and without TGFβ (10 ng/mL) for 1 and 2 days. Results demonstrated a marked increase in relative expression of *Ncf1* but not *Ncf2* or *gp91^phox^* in stimulated cells (**Figure 6**). Samples stimulated with and without TGFβ demonstrated no significant differences in expression indicating that the observed augmentation of ROS by TGFβ was not due to enhanced gene expression. However, the observation that stimulation in general results in a marked increase in *Ncf1* expression may indicate that it has a wider role in TCR/CD28 signaling than has previously been thought.

### Ncf1^−/−^ Tregs are poorly suppressive to Ncf1^−/−^ Teff and there is incomplete reconstitution of suppression of wild type Teff by Ncf1^−/−^ Tregs and of Ncf1^−/−^ Teff by wild type Tregs

**Figure 7:**
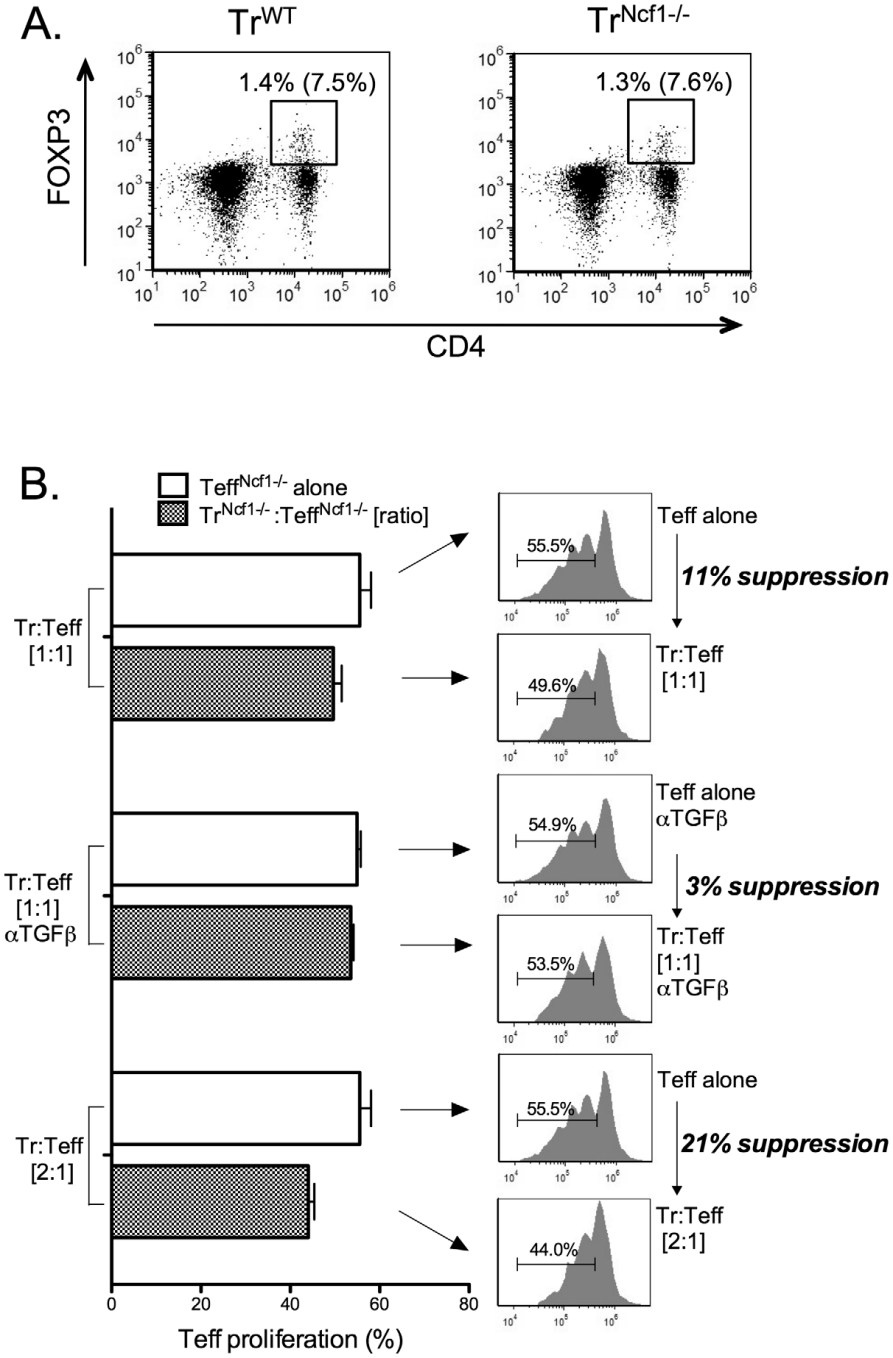
Suppression of *Ncf1^−/−^* Teffs by *Ncf1^−/−^* Tregs is markedly decreased. (A) Tregs were quantified in total spleen cell suspensions from WT C57BL/6 mice (Tr^WT^) and from Ncf1-deficient mice (Tr^Ncf1−/−^) by flow cytometric evaluation of CD4 and FOXP3 expression. CD4+FOXP3+ Tregs are shown gated with the percentage of Tregs in total spleen cells and the percentage of Tregs among only CD4+ T cells in parentheses. This data is representative of that observed from three WT and three *Ncf1^−/−^* mice. (B) Suppression assays were performed using *Ncf1^−/−^* Tregs and *Ncf1^−/−^* Teffs co-cultured at the indicated ratios (1∶1 and 2∶1) along with *Ncf1^−/−^* Teffs alone as controls. TGFβ neutralizing antibodies (30 µg/mL) were added to one set of samples as indicated. Samples were stimulated for 3 days with CD3/CD28 beads. The data shows proliferation of *Ncf1^−/−^* Teffs cultured alone and *Ncf1^−/−^* Teffs co-cultured with *Ncf1^−/−^* Tregs in suppression assays. CFSE staining data is shown at right along with the calculated suppression for this representative result (%). The data shown is representative of at least 3 identical experiments.

Because we observed that NAC, 2-ME and NADPH oxidase inhibitors all resulted in varying degrees of inhibited suppression, and since exogenous TGFβ was less effective at suppressing the proliferation of Ncf1-deficient Teffs, we next evaluated suppression using Tregs and Teffs isolated from *Ncf1^−/−^* mice. First, we evaluated the total number of splenic Tregs in these mice to assess for quantitative differences, due to differential NADPH oxidase function *in vivo*, and found none (**Figure 7A**). Subsequently, we performed suppression assays using *Ncf^−/−^* Tregs (Tregs^Ncf1−/−^) as suppressor cells along with *Ncf^−/−^* Teffs (Teffs^Ncf1−/−^). We found markedly reduced overall suppression at Treg:Teff ratios of both 1∶1 and 2∶1 (**Figure 7B**) as compared to that seen in identical assays using wild type Tregs and Teffs. A 1∶1 ratio yielded a mean suppression of 11.7% (±4.04) and a 2∶1 ratio yielded a mean suppression of 24.3% (±5.77). This compares to suppression levels of approximately 50% for a 1∶1 ratio using wild type cells and >60% for a 2∶1 ratio with wild type cells (see Figure 2B). Interestingly, the addition of TGFβ neutralizing antibodies still had a small but significant effect on blocking suppression (suppression  = 2.3% ±1.2 for a 1∶1 ratio of Tregs:Teff with 30 µg/mL anti-TGFβ), essentially eliminating what residual suppression remained (p = 0.02 for the comparison of a 1∶1 ratio with and without anti-TGFβ). These results show that the absence of Ncf1, and by extension the lack of functional NADPH oxidase, results in markedly reduced, but not completely absent, direct Treg suppression of Teffs.

**Figure 8:**
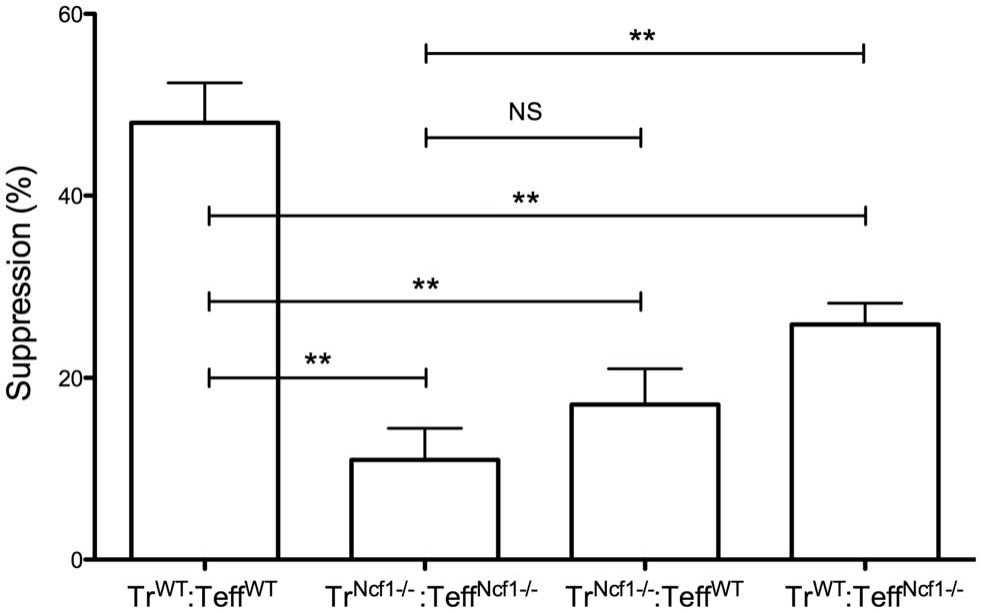
There is significant but incomplete reconstitution of suppression of *Ncf1^−/−^* Teffs by wild type Tregs. Suppression assays were performed with mixtures (all at a 1∶1 ratio) of wild type Tregs and Teffs (labeled Tr^WT^ and Teff ^WT^ respectively) and *Ncf1^−/−^* Tregs and Teffs (labeled Tr^Ncf1-^ and Teff^Ncf1-^ respectively) as indicated in the figure. As above, samples were stimulated for three days with anti-CD3/CD28 beads and suppression was calculated by measuring Teff proliferation when plated alone and when plated with Tregs. The data shown is a compilation of at least three identical experiments, each performed in triplicate and each yielding similar results (NS – not significant, **p≤0.01).

In light of the finding of very poor suppression of Teffs^Ncf1−/−^ by Tregs^Ncf1−/−^ we wondered whether this effect was due to a lack of Ncf1 in the Teffs, as we had initially hypothesized, or whether it could be attributable to both T cell subsets. To assess this, we performed suppression assays using wild type Tregs (Tregs^WT^) co-cultured with CFSE labeled Teffs^Ncf1−/−^ and Tregs^Ncf1−/−^ co-cultured with wild type CFSE-labeled Teffs (Teffs^WT^). In this way, the ramifications of the lack of NADPH oxidase could be isolated to either Tregs or Teffs. Suppression assays performed in this way are shown in **Figure 8**. Conditions with Tregs^Ncf1−/−^ co-cultured with Teffs^WT^ and with Tregs^WT^ co-cultured with Teffs^Ncf1−/−^ showed significantly reduced suppression as compared to control assays with Teffs^WT^ and Tregs^WT^ (p<0.002 for both comparisons). Conditions with Tregs^Ncf1−/−^ and Teffs^WT^ demonstrated slightly higher suppression than control samples with Tregs^Ncf1−/−^ and Teffs^Ncf1−/−^ but this was not significant (p = 0.11). Assays with Tregs^WT^ and Teffs^Ncf1−/−^ demonstrated significantly higher levels of suppression than Tregs^Ncf1−/−^ and Teffs^Ncf1−/−^ (p = 0.003). Taken together, these results indicate that *Ncf1* expression, and by extension NADPH oxidase function, is necessary in *both* Tregs and Teffs for optimum suppression to occur.

## Discussion

The process by which Tregs suppress the activation and proliferation of effector T cells remains relatively poorly understood in spite of intense efforts to understand it. A ROS mediated mechanism to potentiate suppression by Tregs has not been explored. However, there is evidence that NADPH oxidase derived ROS play a general role in immunosuppression. One of the strongest genetic associations with autoimmune arthritis in animal models of the disease is the gene *Ncf1* that, when deleted, leads to low ROS production and autoimmunity [32], [33]. A second observation that supports an immunosuppressive role for ROS is found in the inherited syndrome CGD. CGD patients lack NADPH oxidase function, suffer from impaired immunity due to the inability to mount a phagocyte oxidative burst and have high rates of bacterial infections. However, affected individuals also suffer from increased rates of autoimmune disease including arthritis, autoimmune lung disease and lupus erythematosus [34]. In addition, chronic colitis is a major cause of morbidity in the majority of patients [35]. The etiology of CGD related autoimmunity remains unexplained.

Thymus-derived Tregs are markedly less sensitive than effector T cells to oxidant stress induced cell death [36] and this perhaps suggests a particular role for ROS in Treg function and physiology. *Glutaredoxin*, encoding an enzyme involved in redox regulation and glutathione-mediated oxidant defense, is specifically upregulated by the Treg-specific transcription factor FOXP3 [37]. There is evidence that Tregs may indirectly suppress the proliferation of Teffs by inhibiting the secretion of glutathione/cysteine into the microenvironment by dendritic cells which otherwise would create an extracellular redox potential that facilitates T cell proliferation [18]. Furthermore, a recent study showed that Tregs modulate glutathione metabolism in Teffs via a cell-contact and antigen-dependent, but not antigen-specific, mechanism during suppression [38]. The mechanism for this was not identified but could potentially involve NADPH oxidase. Macrophages have also been shown to suppress T cell activation *in vitro* and *in vivo* through ROS [39] and, recent data demonstrates that macrophages induce Tregs via a ROS dependent pathway that can be blocked by the NADPH oxidase inhibitor apocynin [40]. However, the potential role of TGFβ was not addressed in the latter study. This finding, considered together with the observation that ROS is augmented by TGFβ, highlights the possibility that the induction of Tregs in the periphery by TGFβ could be related to ROS. However, in the case of thymus derived natural Tregs, presumably comprising the majority of spleen Tregs, our data demonstrates no quantitative differences in animals lacking NADPH oxidase function.

Our findings showed that thiol-bearing antioxidants and NADPH oxidase inhibitors had an antagonistic effect on Treg-mediated suppression, supporting the hypothesis that NADPH oxidase derived ROS is important in this process. Furthermore, a lack of Ncf1 in both Tregs *and* Teffs was also associated with markedly reduced suppression that was partially but significantly reconstituted in assays with wild type Tregs suppressing Ncf1-deficient Teff. Taken together, our data indicate that NADPH oxidase function in both Tregs and in targeted effector CD4+ T cells is necessary for suppression. We hypothesize that these effects are mediated only partially by TGFβ receptor signaling in Teffs. Previous findings suggest that T cell derived ROS, produced in response to CD3/CD28 triggering, is necessary for the activation of latent TGFβ [23] and this could possibly explain some of the findings that we have observed. However, the observation that NAC can overcome the suppressive effects of exogenous, active TGFβ shows that this is not a completely sufficient explanation. Furthermore, TGFβ neutralization still results in a decrease in the residual suppression seen with Tregs and Teff, both lacking Ncf1. One explanation would be that TGFβ conditions Teffs to respond to Tregs via NADP oxidase dependent and non-dependent pathways that are necessary but not fully sufficient for suppression to occur. This remains to be seen.

In additional to its apparent importance in Teffs, we also show that NADPH oxidase function in Tregs themselves is a major contributor to the observed suppression. The means by which this occurs are unclear but perhaps Treg derived ROS could serve to alter the redox potential of the microenvironment in a similar fashion to ROS produced by Teff themselves. Furthermore, close apposition of Tregs and Teff could facilitate this process and this may be an additional explanation for the apparently cell contact dependent suppression observed in many *in vitro* studies. Such a mechanism could possibly contribute to the depletion of cysteine and glutathione that has been recently observed in Teffs undergoing suppression by Tregs [38].

The finding that NADPH oxidase function in Tregs is necessary for optimum suppression serves as a possible explanation for why TGFβ neutralizing antibodies are only partially effective in our experiments at inhibiting suppression. Partial blockade of suppression in the setting of TGFβ neutralization has been observed previously but complete blockade was achieved in the presence of both TGFβ and CTLA-4 neutralizing antibodies [4]. However, Tregs from CTLA-4 knockout mice still display normal suppressive function *in vitro* [41]. Thus no single mechanism or pathway has been implicated as being indispensible to the suppressive function of Tregs. We observed that TGFβ neutralizing antibodies still had an effect on suppression in assays with Tregs and Teff both lacking Ncf1. Furthermore, exogenous TGFβ was still suppressive to Ncf1-deficient Teffs. This indicates that TGFβ has suppressive effects in the absence of NADPH oxidase function. *Ncf1^−/−^* mice display features of increased autoimmunity [31], [33], [42] and we have shown that their Tregs are very poorly suppressive *in vitro*. However, these animals certainly do not suffer from the severity of autoimmunity that is seen in mice with *foxp3* mutations and a complete lack of Tregs [43] or in mice that have been depleted of Tregs [44], [45], [46]. Our observations indicate that there is some remaining suppressive function, apparently related to TGFβ, that is displayed during the interaction of *Ncf1^−/−^* Tregs and *Ncf1^−/−^* Teff and perhaps this may serve as an explanation for mild to moderate degree of autoimmunity in *Ncf1^−/−^* mice. The reduced Treg function in these animals may simply predispose to the development of autoimmunity, especially in response to certain stimuli such as those used to experimentally induce arthritis [33]. An apparent predisposition to autoimmunity in *Ncf1^−/−^* mice is similar to the findings in human CGD patients.

In summary, our studies show that suppression by mouse Tregs can be blocked, *in vitro*, by thiol-bearing antioxidants, NADPH oxidase inhibitors or by genetic deficiency in the NADPH oxidase component *Ncf1*, supporting an important role for oxidants and the cellular redox state in Treg-mediated suppression. Furthermore, NADPH oxidase function in *both* Tregs and Teffs appears to be a requirement for optimum suppression in a pathway that partially involves TGFβ. We hypothesize that redox modulation, whether by MDSC, dendritic cells or Tregs, may be a central theme in the regulation of effector T cell function by regulatory immune subsets in a variety of circumstances. Mechanisms of ROS production may represent an attractive target for the development of new immune modulating therapies.

## References

[1] Cao X, Cai SF, Fehniger TA, Song J, Collins LI (2007). Granzyme B and perforin are important for regulatory T cell-mediated suppression of tumor clearance.. Immunity.

[2] Sakaguchi S, Wing K, Onishi Y, Prieto-Martin P, Yamaguchi T (2009). Regulatory T cells: how do they suppress immune responses?. Int Immunol.

[3] Stockis J, Colau D, Coulie PG, Lucas S (2009). Membrane protein GARP is a receptor for latent TGF-beta on the surface of activated human Treg.. Eur J Immunol.

[4] Oida T, Xu L, Weiner HL, Kitani A, Strober W (2006). TGF-beta-mediated suppression by CD4+CD25+ T cells is facilitated by CTLA-4 signaling.. J Immunol.

[5] Savage ND, de Boer T, Walburg KV, Joosten SA, van Meijgaarden K (2008). Human anti-inflammatory macrophages induce Foxp3+ GITR+ CD25+ regulatory T cells, which suppress via membrane-bound TGFbeta-1.. J Immunol.

[6] Fahlen L, Read S, Gorelik L, Hurst SD, Coffman RL (2005). T cells that cannot respond to TGF-beta escape control by CD4(+)CD25(+) regulatory T cells.. J Exp Med.

[7] Christ M, McCartney-Francis NL, Kulkarni AB, Ward JM, Mizel DE (1994). Immune dysregulation in TGF-beta 1-deficient mice.. J Immunol.

[8] Neurath MF, Fuss I, Kelsall BL, Presky DH, Waegell W (1996). Experimental granulomatous colitis in mice is abrogated by induction of TGF-beta-mediated oral tolerance.. J Exp Med.

[9] Yoshimura A, Wakabayashi Y, Mori T (2010). Cellular and molecular basis for the regulation of inflammation by TGF-beta.. J Biochem.

[10] Zheng SG, Wang J, Wang P, Gray JD, Horwitz DA (2007). IL-2 is essential for TGF-beta to convert naive CD4+CD25- cells to CD25+Foxp3+ regulatory T cells and for expansion of these cells.. J Immunol.

[11] Bondi CD, Manickam N, Lee DY, Block K, Gorin Y (2010). NAD(P)H oxidase mediates TGF-beta1-induced activation of kidney myofibroblasts.. J Am Soc Nephrol.

[12] Boudreau HE, Emerson SU, Korzeniowska A, Jendrysik MA, Leto TL (2009). Hepatitis C virus (HCV) proteins induce NADPH oxidase 4 expression in a transforming growth factor beta-dependent manner: a new contributor to HCV-induced oxidative stress.. J Virol.

[13] Amara N, Goven D, Prost F, Muloway R, Crestani B (2010). NOX4/NADPH oxidase expression is increased in pulmonary fibroblasts from patients with idiopathic pulmonary fibrosis and mediates TGFbeta1-induced fibroblast differentiation into myofibroblasts.. Thorax.

[14] Meurer SK, Lahme B, Tihaa L, Weiskirchen R, Gressner AM (2005). N-acetyl-L-cysteine suppresses TGF-beta signaling at distinct molecular steps: the biochemical and biological efficacy of a multifunctional, antifibrotic drug.. Biochem Pharmacol.

[15] Felton VM, Borok Z, Willis BC (2009). N-acetylcysteine inhibits alveolar epithelial-mesenchymal transition.. Am J Physiol Lung Cell Mol Physiol.

[16] Hadzic T, Li L, Cheng N, Walsh SA, Spitz DR (2005). The role of low molecular weight thiols in T lymphocyte proliferation and IL-2 secretion.. J Immunol.

[17] Edinger AL, Thompson CB (2002). Antigen-presenting cells control T cell proliferation by regulating amino acid availability.. Proc Natl Acad Sci U S A.

[18] Yan Z, Garg SK, Kipnis J, Banerjee R (2009). Extracellular redox modulation by regulatory T cells.. Nat Chem Biol.

[19] Srivastava MK, Sinha P, Clements VK, Rodriguez P, Ostrand-Rosenberg S (2010). Myeloid-derived suppressor cells inhibit T-cell activation by depleting cystine and cysteine.. Cancer Res.

[20] Gelderman KA, Hultqvist M, Holmberg J, Olofsson P, Holmdahl R (2006). T cell surface redox levels determine T cell reactivity and arthritis susceptibility.. Proc Natl Acad Sci U S A.

[21] Jackson SH, Devadas S, Kwon J, Pinto LA, Williams MS (2004). T cells express a phagocyte-type NADPH oxidase that is activated after T cell receptor stimulation.. Nat Immunol.

[22] Devadas S, Zaritskaya L, Rhee SG, Oberley L, Williams MS (2002). Discrete generation of superoxide and hydrogen peroxide by T cell receptor stimulation: selective regulation of mitogen-activated protein kinase activation and fas ligand expression.. J Exp Med.

[23] Amarnath S, Dong L, Li J, Wu Y, Chen W (2007). Endogenous TGF-beta activation by reactive oxygen species is key to Foxp3 induction in TCR-stimulated and HIV-1-infected human CD4+CD25- T cells.. Retrovirology.

[24] Kwon J, Devadas S, Williams MS (2003). T cell receptor-stimulated generation of hydrogen peroxide inhibits MEK-ERK activation and lck serine phosphorylation.. Free Radic Biol Med.

[25] Huang CK, Zhan L, Hannigan MO, Ai Y, Leto TL (2000). P47(phox)-deficient NADPH oxidase defect in neutrophils of diabetic mouse strains, C57BL/6J-m db/db and db/+.. J Leukoc Biol.

[26] Hancock JT, Jones OT (1987). The inhibition by diphenyleneiodonium and its analogues of superoxide generation by macrophages.. Biochem J.

[27] ten Freyhaus H, Huntgeburth M, Wingler K, Schnitker J, Baumer AT (2006). Novel Nox inhibitor VAS2870 attenuates PDGF-dependent smooth muscle cell chemotaxis, but not proliferation.. Cardiovasc Res.

[28] Lyons AB (1999). Divided we stand: tracking cell proliferation with carboxyfluorescein diacetate succinimidyl ester.. Immunol Cell Biol.

[29] Pfaffl MW (2001). A new mathematical model for relative quantification in real-time RT-PCR.. Nucleic Acids Res.

[30] Jackson SH, Gallin JI, Holland SM (1995). The p47phox mouse knock-out model of chronic granulomatous disease.. J Exp Med.

[31] Hagenow K, Gelderman KA, Hultqvist M, Merky P, Backlund J (2009). Ncf1-associated reduced oxidative burst promotes IL-33R+ T cell-mediated adjuvant-free arthritis in mice.. J Immunol.

[32] Olofsson P, Holmberg J, Tordsson J, Lu S, Akerstrom B (2003). Positional identification of Ncf1 as a gene that regulates arthritis severity in rats.. Nat Genet.

[33] Hultqvist M, Olofsson P, Holmberg J, Backstrom BT, Tordsson J (2004). Enhanced autoimmunity, arthritis, and encephalomyelitis in mice with a reduced oxidative burst due to a mutation in the Ncf1 gene.. Proc Natl Acad Sci U S A.

[34] De Ravin SS, Naumann N, Cowen EW, Friend J, Hilligoss D (2008). Chronic granulomatous disease as a risk factor for autoimmune disease.. J Allergy Clin Immunol.

[35] Huang A, Abbasakoor F, Vaizey CJ (2006). Gastrointestinal manifestations of chronic granulomatous disease.. Colorectal Dis.

[36] Mougiakakos D, Johansson CC, Kiessling R (2009). Naturally occurring regulatory T cells show reduced sensitivity toward oxidative stress-induced cell death.. Blood.

[37] Sugimoto N, Oida T, Hirota K, Nakamura K, Nomura T (2006). Foxp3-dependent and -independent molecules specific for CD25+CD4+ natural regulatory T cells revealed by DNA microarray analysis.. Int Immunol.

[38] Yan Z, Garg SK, Banerjee R (2010). Regulatory T cells interfere with glutathione metabolism in dendritic cells and T cells.. J Biol Chem.

[39] Gelderman KA, Hultqvist M, Pizzolla A, Zhao M, Nandakumar KS (2007). Macrophages suppress T cell responses and arthritis development in mice by producing reactive oxygen species.. J Clin Invest.

[40] Kraaij MD, Savage ND, van der Kooij SW, Koekkoek K, Wang J (2010). Induction of regulatory T cells by macrophages is dependent on production of reactive oxygen species.. Proc Natl Acad Sci U S A.

[41] Tang Q, Boden EK, Henriksen KJ, Bour-Jordan H, Bi M (2004). Distinct roles of CTLA-4 and TGF-beta in CD4+CD25+ regulatory T cell function.. Eur J Immunol.

[42] van de Loo FA, Bennink MB, Arntz OJ, Smeets RL, Lubberts E (2003). Deficiency of NADPH oxidase components p47phox and gp91phox caused granulomatous synovitis and increased connective tissue destruction in experimental arthritis models.. Am J Pathol.

[43] Godfrey VL, Wilkinson JE, Russell LB (1991). X-linked lymphoreticular disease in the scurfy (sf) mutant mouse.. Am J Pathol.

[44] McHugh RS, Shevach EM (2002). Cutting edge: depletion of CD4+CD25+ regulatory T cells is necessary, but not sufficient, for induction of organ-specific autoimmune disease.. J Immunol.

[45] Itoh M, Takahashi T, Sakaguchi N, Kuniyasu Y, Shimizu J (1999). Thymus and autoimmunity: production of CD25+CD4+ naturally anergic and suppressive T cells as a key function of the thymus in maintaining immunologic self-tolerance.. J Immunol.

[46] Lahl K, Loddenkemper C, Drouin C, Freyer J, Arnason J (2007). Selective depletion of Foxp3+ regulatory T cells induces a scurfy-like disease.. J Exp Med.

